# Genetic characterization and virulence determinants of multidrug-resistant NDM-1-producing *Aeromonas caviae*

**DOI:** 10.3389/fmicb.2022.1055654

**Published:** 2023-01-16

**Authors:** Xinjun Hu, Huanran Zhang, Yi Liu, Xiaojing Liu, Jie Qiao, Haoyu Ge, Junhui Zhao, Xiaohan Ma, Mantao Chen, Ruishan Liu

**Affiliations:** ^1^Department of Infectious Diseases, The First Affiliated Hospital, College of Clinical Medicine, Henan University of Science and Technology, Luoyang, China; ^2^Department of Emergency Medicine, The First Affiliated Hospital, School of Medicine, Zhejiang University, Hangzhou, China; ^3^Key Laboratory for Diagnosis and Treatment of Aging and Physic-Chemical Injury Diseases of Zhejiang Province, Hangzhou, China; ^4^State Key Laboratory for Diagnosis and Treatment of Infectious Diseases, Collaborative Innovation Center for Diagnosis and Treatment of Infectious Diseases, The First Affiliated Hospital, College of Medicine, Zhejiang University, Hangzhou, China; ^5^Department of Structure and Morphology, Jinan Microecological Biomedicine Shandong Laboratory, Jinan, China; ^6^School of Basic Medical Sciences, Beihua University, Jilin, China; ^7^Department of Neurosurgery, The First Affiliated Hospital, College of Medicine, Zhejiang University, Hangzhou, China

**Keywords:** *Aeromonas caviae*, *bla*_NDM–1_, whole-genome sequencing, phylogenetic analysis, biofilm formation

## Abstract

The emergence of carbapenemase significantly threatens public health. It is prevalent worldwide but rare in *Aeromonas caviae*. Unlike most bacterial species, *A. caviae* has two distinct flagella systems, which are closely related to biofilm formation. The ability to form biofilms on host tissues or inert surfaces constitutes an important cause of many persistent infections, which causes difficulties in clinical treatment. Here, we report on a multidrug-resistant (MDR) *A. caviae* carrying *bla*_NDM–1_ with a novel sequence type 1,416. The strong ability of biofilm formation of FAHZZU2447 was verified by a crystal violet assay. The resistome profile and location of the *bla*_NDM–1_ gene were determined by antimicrobial susceptibility testing, S1 nuclease pulsed-field gel electrophoresis (S1-PFGE), and Southern blot analysis. Moreover, the strain underwent whole-genome sequencing to identify its genomic characteristics. In addition, the *bla*_NDM–1_ gene was located on a ∼243 kb plasmid with genetic context IS*1R*-*bla*_NDM–1_-*ble*-*trpF*-*dsbD*-*hp*-*sul1*-*qacE*. Phylogenetic analysis indicated the transmission of *A. caviae* in China, Japan, and Thailand. Our study aimed to elucidate the genomic features of *bla*_NDM–1_-producing *A. caviae*, thereby clarifying the distribution of *A. caviae* worldwide and emphasizing the harmfulness of biofilm formation to the clinic. Further comprehensive surveillance of this species is needed to control further dissemination.

## Introduction

*Aeromonas* spp. is ubiquitous in aquatic environments and has been considered a significant human pathogen since it was isolated from a blood sample in 1954 (Parker and Shaw, 2011). *Aeromonas* spp. is a common contaminant of fish and seafood (Hänninen et al., 1997). It usually causes invasive extraintestinal infections, including biliary tract infections, by ingesting food contaminated with *Aeromonas* spp. (Parker and Shaw, 2011). Until now, a total of 36 species have been described in the genus *Aeromonas*, and all cause widespread infections (Fernández-Bravo and Figueras, 2020). However, according to a recent report, the infections caused by *Aeromonas* spp. in the clinic mainly focused on four species: *Aeromonas caviae*, *Aeromonas dhakensis*, *Aeromonas veronii*, and *Aeromonas hydrophila* (Fernández-Bravo and Figueras, 2020). Among them, *A. caviae* was the most common species but rarely causes biliary tract infection (Janda and Abbott, 2010; Chao et al., 2013).

New Delhi metallo-β-lactamase (NDM) is a metallo-β-lactamase that offers carbapenem antibiotic resistance to hydrolyze to almost all beta-lactam antibiotics, except aztreonam, which significantly threatens public health, worldwide (Nordmann et al., 2011). Since NDM-1 was first detected in 2008, in *Klebsiella pneumoniae* from a patient repatriated from India, it has disseminated worldwide (Yong et al., 2009). However, the current spread of NDM-1 was related to Enterobacterales and limited reports are available of NDM-1-carrying *Aeromonas* spp. (Luo et al., 2022; Xu et al., 2022). To date, only two studies have reported on NDM-1-producing *A. caviae*, which were isolated from sputum and blood, and *bla*_NDM–1_ genes were located on the plasmid and chromosome, respectively (Luo et al., 2022; Xu et al., 2022). The emergence of NDM-1-harboring *A. caviae* in the clinic is concerning.

Many bacterial species express either single/multiple polar flagella or peritrichous (lateral non-induced) flagella. Few strains possess dual flagellar systems and express two entirely distinct flagella systems: polar flagellum and lateral flagella for swimming and swarming, respectively (Santos et al., 2011). The swimming motility of *A. caviae* in liquid environments is possible by expressing polar unsheathed monotrichous flagellum (*fla*). Furthermore, *A. caviae* produced inducible lateral flagella (*laf*) when cultivated on solid or viscous media. In addition, the phenomenon is associated with the colonization of surfaces, biomass production, and biofilm formation (Gavín et al., 2003). The ability to form biofilms on host tissues or inert surfaces is an important cause of many persistent infections and causes difficulties in clinical treatment that need our attention (Parsek and Singh, 2003).

In previous studies, epidemiological studies have been conducted on carbapenem-resistant *A. caviae* clinical isolates; however, less attention was paid to biofilm formation. In this study, a multidrug-resistant (MDR) *A. caviae* strain carrying NDM-1 with a new sequence type (ST) 1,416 was isolated. The ability of biofilm formation was verified and compared. In addition, the microbiological and molecular mechanisms involved were elucidated. Furthermore, to elucidate the distribution characteristics of *A. caviae*, comprehensive phylogenetic analyses were conducted.

## Materials and methods

### Isolation and identification of bacteria

*Aeromonas caviae* strain FAHZZU2447 was isolated from a patient with a biliary tract infection and was hospitalized in a tertiary hospital in Zhengzhou, China. The species was identified by matrix-assisted laser desorption/ionization time-of-flight mass spectrometry (MALDI-TOF/MS) (Bruker, Bremen, and Germany) and genome sequence-based average nucleotide identity (ANI) analysis (Richter and Rosselló-Móra, 2009). The *bla*_NDM–1_ gene was detected using PCR analysis and Sanger sequencing. The primers used were as follows: *bla*_NDM–1_-F, 5′- ATGGAATTGCCCAATATTATGCAC-3′; and *bla*_*DNM–*1_-R, 5′- TCAGCGCAGCTTGTCGGC-3′.

### Antimicrobial susceptibility testing

The bacterial resistance was determined using both broth microdilution and agar dilution methods, and the results QQwere interpreted according to the Clinical and Laboratory Standards Institute [CLSI], 2021^1^ and European Committee on Antimicrobial Susceptibility Testing (EUCAST) clinical breakpoints.^2^ *Escherichia coli* ATCC 25922 and *Pseudomonas aeruginosa* ATCC 27853 served as quality controls. The antibiotics tested in this study included piperacillin/tazobactam, ceftazidime, ceftriaxone, cefepime, cefotaxime, ciprofloxacin, imipenem, trimethoprim/sulfamethoxazole, amikacin, gentamicin, aztreonam, chloramphenicol, tetracycline, colistin, and tigecycline.

### Location of *bla*_NDM–1_ gene and transferability of plasmids carrying *bla*_NDM–1_

The size and number of plasmids of strain FAHZZU2447 were confirmed using S1 nuclease-pulsed field gel electrophoresis (S1-PFGE) (Xu et al., 2018). Using a digoxigenin-labeled *bla*_NDM–1_ probe, Southern blot analysis was conducted to verify the location of the *bla*_NDM–1_ gene. Furthermore, a conjugation assay was performed using *E. coli* J53 as the recipient strain to test the transferability of the plasmid carrying NDM-1. Next, transconjugants were selected on agar (OXOID, Hampshire, UK) medium, containing 200 mg/L sodium azide and 2 mg/L meropenem. The verification of transconjugants was carried out by both MALDI-TOF/MS and PCR analysis.

### Whole-genome sequencing and bioinformatics analysis

The complete sequence of FAHZZU2447 was obtained using whole-genome sequencing (WGS). Briefly, DNA was extracted using Gentra Puregene Yeast/Bact. Kit (Qiagen, Dusseldorf, Germany) and then sequenced on Illumina Novaseq 6000 (Illumina, San Diego, CA, USA) and Oxford Nanopore (Oxford Nanopore Technologies, Oxford, UK) platforms. The hybrid assembly was conducted by Unicycler v0.4.7 (Wick et al., 2017). Multilocus sequence typing (MLST) and virulence genes were identified by pubMLST and VFDB 2022 databases, respectively (Jolley et al., 2018; Liu et al., 2022). Finally, the annotation and bioinformatic analysis were performed using Prokka v1.14.0^3^ and an online tool,^4^ respectively. The complete genome sequence of *A. caviae* FAHZZU2447 was uploaded to NCBI with the following accession numbers: CP100392-CP100394.

FAHZZU2447 lacks plasmid-mediated quinolone resistance (PMQR) but showed resistance to ciprofloxacin, and the quinolone-resistance-determining region (QRDR) of *gyrA* and *parC* genes was examined for mutations (Yang et al., 2017). Briefly, the QRDR of the *gyrA* and *parC* genes was compared with the sequences of the *A. caviae* complex (GenBank accession numbers: AY027899 and AF435418, respectively) (Arias et al., 2010). Next, OriTFinder was used to predict the complete conjugative modules on a plasmid (Li et al., 2018). Furthermore, the genetic environment surrounding the *bla*_NDM–1_ gene was characterized using easyfig v2.2.5 (Sullivan et al., 2011). The comparison map of plasmids in this study was generated using Brig v0.95 (Alikhan et al., 2011) and compared with related plasmids in the National Center for Biotechnology Information (NCBI) database.

### Motility assays and biofilm formation

The motility of FAHZZU2447 was assessed according to a previous report with minor modifications (Gavín et al., 2002). Briefly, a freshly grown single colony was inoculated into the center of motility agar [0.3% agar in Luria-Bertani broth (OXOID, Hampshire, UK)] and incubated at 37°C for 16–24 h. Next, the motility was assessed by examining the migration of bacteria from the center of the agar toward the periphery of the plate.

Biofilm formation was quantitatively determined through a modified biofilm assay that was based on a previous report (Santos et al., 2011). Briefly, the overnight culture was diluted in LB and dispensed in a 96 well plate (200 μl/well). Wells with only LB broth served as the control. After incubation for 24 h at 37°C without shaking, wells were washed three times with phosphate-buffered saline (PBS) to remove non-adherent bacteria. Next, methanol was added to each well to fix the bacteria, and a 0.1% crystal violet solution was used for staining. After washing three times with PBS, absolute ethanol was added to each well and the optical density (OD) was measured at 595 nm. The cutoff OD (ODc) was defined as the mean OD of the control. The following conditions were used to interpret the results (Christensen et al., 1985): non-adherent (OD ≤ ODc), weakly to moderately adherent (ODc < OD ≤ 2 × ODc), and strongly adherent (2 × ODc < OD). Each assay was performed in triplicate. One-way ANOVA was used for statistical analysis. *P* < 0.05 was considered statistically significant.

### Phylogenetic analysis

To evaluate the distribution characteristics of *A. caviae*, 150 available genomes were downloaded from the NCBI database. To exclude confounding strains, ANI analysis was performed by pyani v0.2.11^5^ (Supplementary Figure 1). Phylogenetic analyses were performed using Roary, and a maximum likelihood phylogenetic tree was constructed with MEGA 11 (Page et al., 2015).

## Results and discussion

### Strain identification and case description

Strain *A. caviae*, designated FAHZZU2447, was isolated from a 67-year-old female patient who was admitted to a tertiary teaching hospital in 2019 in Zhengzhou, China. The patient was admitted for complaints of unexplained nausea and vomiting. The patient underwent cholecystitis resection without postoperative suture removal and biliary T tube drainage. Nausea and vomiting developed after eating dates and cucumbers 7 days earlier, and the vomit contained stomach contents accompanied by abdominal distension. On the day of admission, the patient developed hyperkalemia and underwent dialysis treatment, and piperacillin-tazobactam combined with moxifloxacin was used for anti-infection treatment. On day 7, the patient developed acute kidney failure, and on this day, FAHZZU2447 was isolated from bile. Subsequently, using both MALDI-TOF/MS and ANI analysis, FAHZZU2447 was identified as *A. caviae* (Supplementary Figure 1). Unfortunately, the patient was diagnosed with hepatorenal function and electrolyte disturbance on day 28, and the patient’s family refused further treatment. On day 31, the dialysis tube was removed, and the patient was discharged home.

### Resistome of *A. caviae* FAHZZU2447

Based on the antimicrobial susceptibility testing (AST) results, FAHZZU2447 had a broad drug resistance spectrum and was regarded as an MDR bacterium (Table 1). FAHZZU2447 was resistant to most of the tested antibiotics except for amikacin, tigecycline, chloramphenicol, and colistin. According to the ResFinder database results, FAHZZU2447 harbored plenty of antibiotic resistance genes (ARGs) and mediated resistance to multiple agents of antibiotics (Table 1). Furthermore, the carbapenem resistance gene *bla*_NDM–1_ was identified, which is rarely present in *A. caviae*. Currently, two reports related to *bla*_NDM–1_-harboring *A. caviae* are available and both were isolated from the clinic in China. However, they were not associated with a biliary tract infection (Luo et al., 2022; Xu et al., 2022). In addition, FAHZZU2447 carries other drug-resistance genes, including beta-lactams (*bla*_*TEM–*1*B*_, *bla*_NDM–1_, *bla*_*MOX–*6_, and *bla*_*OXA–*18_), aminoglycosides [*aac(3)-IId*, *aph(6)-Id*, *aph(3′′)-Ib*], tetracyclines [*tet(A)*], phenicols (*catA1*), and sulfonamide (*dfrA5*, *sul1*, *sul2*). Therefore, the resistant phenotype of FAHZZU2447 may mainly be due to the presence of ARGs, except for the resistance to ciprofloxacin, which is associated with mutations in QRDRs. In this study, a total of 83 mutations in *gyrA* codons and 80 mutations in *parC* codons were identified. In addition, substitutions Ser-83-Ile and Ser-87-Ile in *gyrA* and *parC* were found, respectively. As previously described, the mutations at residue 83 of *gyrA* and 87 of *parC* are most frequently encountered and confer a significant increase in the level of quinolone resistance (Goñi-Urriza et al., 2002). However, the substitution in *parC* codon 87 is rare, and this mutation in *A. veronii* strains was only described in one study where it was found to contribute to a higher MIC when co-carrying mutations in *gyrA* codon 83 (Yang et al., 2017).

**TABLE 1:** Susceptibility of *Aeromonas caviae* FAHZZU2447.

Antibiotics	MIC values (μg/mL)	Antimicrobial susceptibility	Mechanism of resistance
Penicillins			-
Piperacillin/tazobactam*^a^*	>128/4	R	
Beta-lactam			*bla*_TEM–1B_, *bla*_NDM–1_, *bla*_MOX–6_, *bla*_OXA–18_
Ceftazidime	>128	R	
Ceftriaxone	>128	R	
Cefepime	128	R	
Cefotaxime	>128	R	
Imipenem	8	R	
Aztreonam	32	R	
Fluoroquinolones			-
Ciprofloxacin	16	R	
Aminoglycosides			*aac(3)-IId*, *aph(6)-Id*, *aph(3″)-Ib*
Amikacin	4	S	
Gentamicin	128	R	
Tetracyclines			*tet(A)*
Tigecycline	0.5	S	
Tetracycline	16	R	
Phenicols			*catA1*
Chloramphenicol	8	S	
Polymyxin			–
Colistin	2	I	
Sulfonamide			*dfrA5*, *sul1*, *sul2*
Trimethoprim/sulfamethoxazole	>8/152	R	
Others			*qacE*, *mph(A)*
Not included in the AST panel	Na	Na	

^*a*^Tazobactam at a fixed concentration of 4 mg/L. R, resistant; I, intermediary; S, susceptible; NA, not applicable.

### Toxome and biofilm formation capacity

As shown in Table 2, FAHZZU2447 containing virulence genes encoded various functions. In previous studies, it has been indicated that the polar flagellum plays a significant role in bacterial adherence, the initial step that precedes colonization (Rabaan et al., 2001). Migration in the surface mediated by lateral flagella permits fast and local colonization, thereby allowing bacteria to multiply and form microcolonies (Lynch et al., 2002; Santos et al., 2011). Thus, flagella-mediated motility is essential for biofilm formation (Kirov et al., 2004). In addition, biofilm formation is a multifactorial process that involves both pili and flagella (Kirov et al., 2004). Type IV pili (T4P), confirmed to be present in *A. caviae*, can substitute for flagellar roles in biofilm formation (Béchet and Blondeau, 2003). Furthermore, the structure MshA pili also seem to play a significant role in biofilm formation by other species, such as *P. aeruginosa* (Santos et al., 2011). FAHZZU2447 carries multiple virulence genes involved in biofilm formation, including *fla*, *laf*, *fli*, *flg*, *che*, and *Tap* genes. Therefore, we hypothesize that FAHZZU2447 has the ability to form biofilms. The motility of FAHZZU2447 was verified (Supplementary Figure 2), and simultaneously, its biofilm formation capacity was assessed using crystal violet. Biofilms were compared with two other NDM-1-carrying *A. caviae* strains as described in our previous study, which carried fewer virulence genes (Xu et al., 2022).

**TABLE 2:** Virulence genes in *Aeromonas caviae* FAHZZU2447.

Functions	Virulence factors	Related genes	Location
Adherence	Lateral flagella	*flgCEIJ, fliFGP, lafBCEFKSTUX, lfgABFGHKLMN, lfhAB, lfiEHIJMNQR, maf-5*	
	Mannose-sensitive hemagglutinin (Msh) pilus, type IV pili	*mshABCDEFG1IJKLMNOP*	
	Polar flagella	*cheABRVWYZ, flaBHJ, flgABCDEFGHIJKLMN, flhABFG, fliAEFGHIJKLNOPQR, flrABC, maf-1, motXY, nueB, pomA2AB2B*	Chromosome
	Tap type IV pili	*TapBCDFMNOPQTUVWY1, tppABCDE*	
Secretion system	T2SS	*exeABCDEFGHIJKLMN*	
	T6SS	*atsD*	
Toxin	Hemolysin HlyA	*hlyA*	
Stress adaptation	Catalase-peroxidase	*katG*	

**FIGURE 1:**
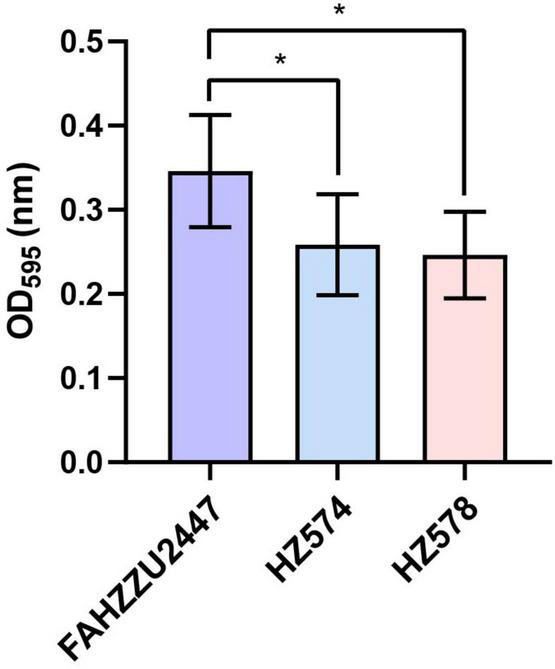
Biofilm formation ability of *Aeromonas caviae* FAHZZU2447, HZ574, and HZ578. Bar charts with purple, blue, and pink represent strains FAHZZU2447, HZ574, and HZ578, respectively. The values shown are the mean ± SD of three independent experiments. *p < 0.05.

The mean OD_595_ values of biofilms of the control, FAHZZU2447, HZ574, and HZ578 were 0.11 ± 0.02, 0.35 ± 0.07, 0.26 ± 0.06, and 0.25 ± 0.05, respectively. All three strains were classified as strongly adherent. Notably, the biofilm formation ability of FAHZZU2447 was stronger than that of the other two strains (*p* < 0.05, Figure 1). The difference in bioform formation ability may be explained by FAHZZU2447 carrying more biofilm formation-related factors, such as lateral flagella and polar flagella (Xu et al., 2022).

### Genomic features of *A. caviae* FAHZZU2447

**FIGURE 2:**
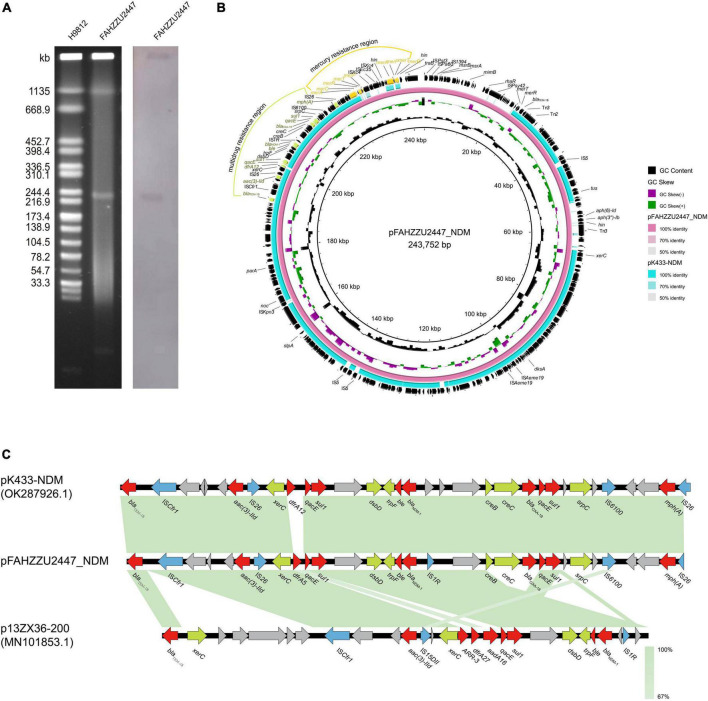
The genetic features of the pFAHZZU2447_NDM plasmid. **(A)** Plasmid profiles of *Aeromonas caviae* FAHZZU2447. **(B)** Comparison of the plasmid pFAHZZU2447_NDM with the closely related plasmid pK433-NDM (accession number: OK287926.1). **(C)** Genetic context of bla_NDM–1_ on pFAHZZU2447_NDM, pK433-NDM, and p13ZX36-200 (accession number: MN101853.1). Different color arrows represent different putative functions. Regions with a high degree of homology are indicated in green.

According to pubMLST, FAHZZU2447 was assigned to a new sequence type ST1416 (*gyrB*-*groL*-*gltA*-*metG*-*ppsA*-*recA*: 96-100-370-650-398-173). S1-PFGE and Southern blot analysis confirmed that the *bla*_NDM–1_ gene was located on the ∼243 kb plasmid pFAHZZU2447_NDM (Figure 2A). Furthermore, WGS data showed that FAHZZU2447 consists of a 4,540,521 bp chromosome with a GC content of 61.5% and two plasmids of 243,752, and 8,061 bp. *In silico* analysis indicated that plasmid pFAHZZU2447_NDM could not be categorized into known replicon types. In addition, NCBI BLAST analysis revealed that pFAHZZU2447_NDM exhibited 99% nucleotide identity with plasmid pK433-NDM (accession number: OK287926.1), which was from clinical strain *A. caviae* K433 in China (Figure 2B). Similar to pFAHZZU2447, pK433-NDM had an unclear plasmid type, and the two plasmids shared a similar backbone. As shown in Figure 2B, the *bla*_NDM–1_ gene is in a multidrug resistance region (MRR) with multiple ARGs and insert sequence (IS) elements (IS*Cfr1*, IS*26*. IS*1R*, and IS*6100*). However, compared with pK433-NDM, pFAHZZU2447_NDM contains an additional mercury resistance region and encodes more ARG and ISs on the plasmid. Genes encoding a small multidrug resistance (SMR) efflux transporter were also found in the MRR, which were deduced to be associated with the efflux system (Kazama et al., 1998; Shen et al., 2021). Furthermore, both pK433-NDM and pFAHZZU2447_NDM lacked a *tra* module, which encodes a primary pilus for conjugation (Chi et al., 2020). In addition, the OriTFinder results indicated that pFAHZZU2447_NDM had incomplete conjugative modules with the absence of a transfer site (*oriT*) and type IV coupling protein (T4CP) (Supplementary Table 1). To verify the transferability of pFAHZZU2447_NDM, conjugation experiments were conducted. However, repeated transformation methods failed, which implied that it was non-conjugative. Moreover, the genetic context of *bla*_NDM–1_ in pFAHZZU2447_NDM (IS*1R*-*bla*_NDM–1_-*ble*-*trpF*-*dsbD*-*hp*-*sul1*-*qacE*) was identical to pK433-NDM and p13ZX36-200 (Figure 2C, accession number: MN101853.1). Especially, two copies of IS*26* elements surrounding the *bla*_NDM–1_-harboring MDR region formed a composite transposon-like structure, which promoted its transfer among various plasmids (Li et al., 2021). The diversity of genetic elements leads to the wide spread of ARGs among bacteria, which needs further attention.

### Analysis of phylogenetic relationships

**FIGURE 3:**
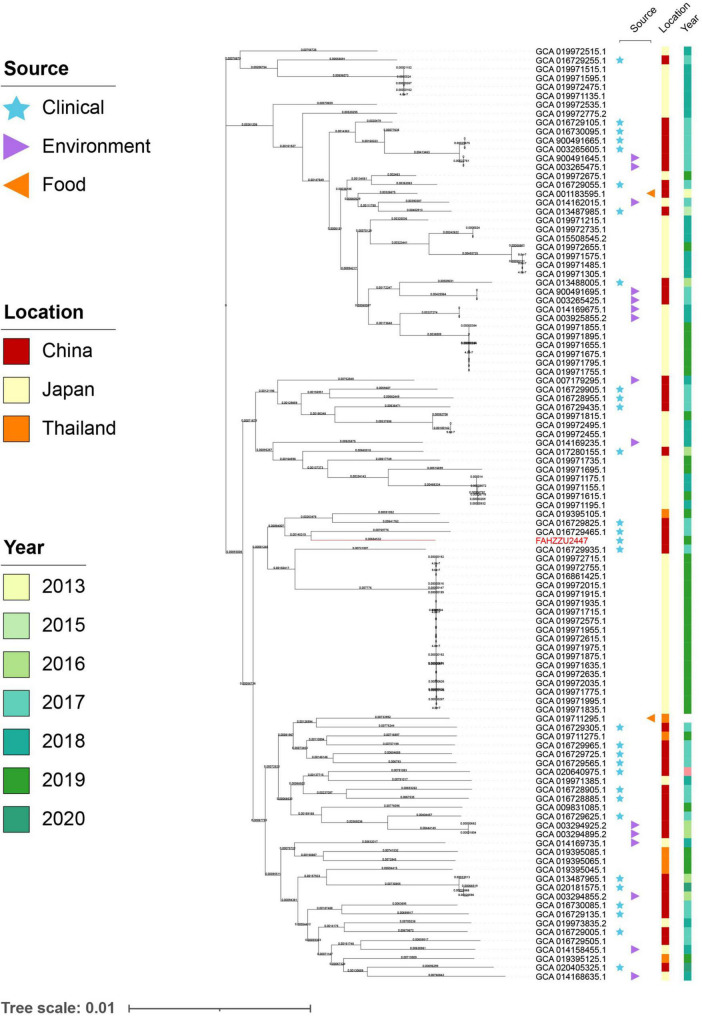
The maximum likelihood core-gene phylogenetic tree of *Aeromonas caviae*. The isolation sources, locations, and collection dates of isolates are shown. Blue stars, purple triangles, and orange triangles represent clinical, environmental, and food origins, respectively; the red, orange, and yellow squares represent strains isolated from China, Japan, and Thailand; the light to dark green colors represent different collection times. *A. caviae* FAHZZU2447 is indicated in red.

To investigate the distribution of *A. caviae* at the global level, a total of 150 available genomes were downloaded from NCBI (Supplementary Table 2). Among these, 139 genomes had a definite source of isolation (including FAHZZU2447), distributed in five continents. Notably, the majority were isolated from Asia (110/139, 79.14%), followed by North America (11/139, 7.91%), South America (9/139, 6.47%), Europe (6/139, 4.32%), and Africa (3/139, 2.16%). Further analysis of the Asian-derived strains showed that transmissions mainly occurred in China, Japan, and Thailand (Supplementary Figure 3). Therefore, the maximum likelihood phylogenetic tree of 105 *A. caviae* isolates from China, Japan, and Thailand was constructed. As shown in Figure 3, the closest relative of FAHZZU2447 is *A. caviae* Colony274 (GCA 019711295.1) from Thailand. Additionally, strains from China and Thailand (GCA 016729305.1 and GCA 019711295.1), as well as from China and Japan (GCA 016729055.1 and GCA 019972675.1), were closely related, thereby indicating the dissemination of *A. caviae* among countries. *A. caviae* are widespread in aquatic creatures and have been isolated from a variety of seafood. Ingesting seafood has caused *A. caviae* infections (Wu et al., 2019). In a previous survey conducted in Taiwan, it was discovered that *Aeromonas* isolates contaminated 88% of seafood from markets, and 33% of the *A. caviae* produced beta-hemolysin (Wu et al., 2019). Japan and Thailand are the primary aquatic product trading countries of China (Liu and Chen, 2011; Yao et al., 2017). Thus, the trade may have accelerated the spread of *A. caviae*. Figure 3 shows that most strains were isolated from the clinic, which is a reminder that continuous monitoring is required.

## Conclusion

In summary, in this study, a *bla*_NDM–1_-carring *A. caviae* FAHZZU2447 from biliary tract infection is reported. FAHZZU2447 harbored various virulence genes and had strong biofilm formation ability, which contributes to persistent infections in the clinic. WGS data indicated that the *bla*_NDM–1_ gene is located in a multidrug resistance region with various ISs. The phylogenetic analysis shows that the transmission of *A. caviae* mainly occurred in China, Japan, and Thailand. Therefore, continuous monitoring and investigations of *A. caviae* are of utmost importance.

## Data availability statement

The datasets presented in this study can be found in online repositories. The names of the repository/repositories and accession number(s) can be found in this article/Supplementary material.

## Ethics statement

Written informed consent was obtained from the individual(s) for the publication of any potentially identifiable images or data included in this article.

## Author contributions

XH, MC, and RL conceived and designed the experiments. XH, HZ, YL, and JQ collected samples and performed the experiments. XL, HG, JZ, and XM analyzed the data. XH and RL wrote the manuscript. All authors contributed to the article and approved the submitted version.

## Footnotes

1
https://clsi.org

2
https://www.eucast.org/

3
https://github.com/tseemann/prokka

4
www.genomicepidemiology.org

5
https://github.com/widdowquinn/pyani
