# Supplementary material for: Genetic characterization and virulence determinants of multidrug-resistant NDM-1-producing Aeromonas caviae
Source: Front Microbiol. 2023 Jan 16;13:1055654. doi: 10.3389/fmicb.2022.1055654 (PMC9885098; doi:10.3389/fmicb.2022.1055654)
Supplement: Supplementary file 1 [file Data_Sheet_1.PDF]

Supplementary Material

Table S1 The prediction of conjugative modules in plasmid pFAHZZU2447_NDM

**Type**	**Location (bp)**
oriT	-
Relaxase	44660-46522
T4SS gene cluster	32307-43777, 126917-139188
T4CP	-

Abbreviations: *oriT*, the origin of transfer site; T4SS, type IV secretion system; T4CP, type IV coupling protein.

Table S2 Characteristics of 151 *A. caviae* genome sequences analyzed in this study. The data were downloaded from the NCBI database

Isolate	Location	Source	Year
GCA 009831085.1	China	Clinical	2019
GCA 000820265.2	Unknown	Unknown	Unknown
GCA 016729625.1	China	Clinical	2017
GCA 003857325.1	Brazil	Clinical	2004
GCA 003294925.2	China	Environment	2016
GCA 003294895.2	China	Environment	2016
GCA 020640975.1	China	Clinical	2020

GCA 003322775.1	Afghanistan	Animals	2015
GCA 016728865.1	Brazil	Environment	2013
GCA 016598815.1	Brazil	Environment	2013
GCA 016728905.1	China	Clinical	2017
GCA 016728885.1	China	Clinical	2017
GCA 013282845.1	Poland	Environment	2018
GCA 009906325.1	Unknown	Environment	Unknown
GCA 016126815.2	Brazil	Environment	2013
GCA 001730205.1	Brazil	Animals	2007
GCA 019972515.1	Japan	Unknown	2018
GCA 009905865.1	Unknown	Clinical	Unknown
GCA 000208825.1	Unknown	Unknown	Unknown
GCA 901202955.1	UK	Clinical	Unknown
GCA 021440765.1	Portugal	Food	2019
GCA 009906375.1	Unknown	Clinical	Unknown
GCA 009906335.1	Unknown	Clinical	Unknown
GCA 013488005.1	China	Clinical	2016
GCA 900491695.1	China	Environment	2017
GCA 003265425.1	China	Environment	2017
GCA 019972775.2	Japan	Unknown	2018

GCA 019972535.1	Japan	Unknown	2018
GCA 001183595.1	China	Food	2013
GCA 014162015.1	Japan	Environment	2017
GCA 900407335.1	Tanzania	Clinical	2015
GCA 013487985.1	China	Clinical	2015
GCA 000783695.2	USA	Clinical	2013
GCA 019972675.1	Japan	Unknown	2019
GCA 016729055.1	China	Clinical	2017
GCA 016729105.1	China	Clinical	2017
GCA 016730095.1	China	Clinical	2017
GCA_900491665.1	China	Clinical	2017
GCA 003265605.1	China	Clinical	2017
GCA 900491645.1	China	Environment	2017
GCA 003265475.1	China	Environment	2017
GCA 001270765.1	Mexico	Clinical	2013
GCA 019972735.1	Japan	Unknown	2018
GCA 015508545.2	Japan	Unknown	2018
GCA 019972655.1	Japan	Unknown	2019
GCA 019971575.1	Japan	Unknown	2018
GCA_019971485.1	Japan	Unknown	2018
GCA 019971305.1	Japan	Unknown	2018

GCA 019971215.1	Japan	Unknown	2018
GCA 014169675.1	Japan	Environment	2018
GCA 003925855.2	Japan	Environment	2018
GCA 019971855.1	Japan	Unknown	2019
GCA 019971675.1	Japan	Unknown	2019
GCA 019971795.1	Japan	Unknown	2019
GCA 019971755.1	Japan	Unknown	2019
GCA 019971895.1	Japan	Unknown	2019
GCA 019971655.1	Japan	Unknown	2019
GCA 019711275.1	Thailand	Unknown	2019
GCA 018359875.1	Spain	Clinical	2000
GCA 016729305.1	China	Clinical	2017
GCA 019711295.1	Thailand	Food	Unknown
GCA 018359825.1	Spain	Clinical	1997
GCA 016729965.1	China	Clinical	2017
GCA 016729565.1	China	Clinical	2017
GCA 001730215.1	Brazil	Environment	2006
GCA 004024195.1	USA	Environment	2017
GCA 003849745.1	Brazil	Environment	2004
GCA 901212305.1	UK	Clinical	Unknown

GCA 008802295.1	South Africa	Environment	2017
GCA 016729725.1	China	Clinical	2017
GCA 902388085.1	Unknown	Clinical	Unknown
GCA 000959705.2	Brazil	Clinical	2010
GCA 019971385.1	Japan	Unknown	2018
GCA 016729255.1	China	Clinical	2017
GCA 004024185.1	USA	Environment	2017
GCA 019971515.1	Japan	Unknown	2018
GCA 019971595.1	Japan	Unknown	2018
GCA 019972475.1	Japan	Unknown	2018
GCA 019971135.1	Japan	Unknown	2018
GCA 004024325.1	USA	Environment	2017
GCA 004024495.1	USA	Environment	2017
GCA 004024475.1	USA	Environment	2017
GCA 019395085.1	Thailand	Unknown	2019
GCA 019395065.1	Thailand	Unknown	2019
GCA 014169735.1	Japan	Environment	2018
GCA 001702475.1	India	Clinical	2015
GCA 018359805.1	Spain	Clinical	2008
GCA 019395045.1	Thailand	Unknown	2019
GCA 016730085.1	China	Clinical	2017

GCA 016729135.1	China	Clinical	2017
GCA 006243135.1	Unknown	Animals	Unknown
GCA 013487965.1	China	Clinical	2016
GCA 020181575.1	China	Clinical	2020
GCA 003294855.2	China	Environment	2016
GCA 019973835.2	Japan	Unknown	2018
GCA 016729005.1	China	Clinical	2017
GCA 012102435.1	Brazil	Clinical	2019
GCA 001030105.1	USA	Clinical	2014
GCA 000783715.2	USA	Clinical	2013
GCA 020405325.1	China	Clinical	2020
GCA 014168635.1	Japan	Environment	2018
GCA 019395125.1	Thailand	Unknown	2019
GCA 000813475.1	Malaysia	Environment	2014
GCA 016729505.1	China	Unknown	2017
GCA 014158455.1	Japan	Environment	2018
GCA 014883895.1	USA	Clinical	2013
GCA 000783775.2	USA	Clinical	2013
GCA 018360005.1	Unknown	Clinical	1984
GCA 003350165.1	Unknown	Unknown	Unknown

GCA 900476005.1	Unknown	Unknown	1900/1989
GCA 000819785.1	Unknown	Unknown	Unknown
GCA 007179295.1	China	Environment	2018
GCA 016729905.1	China	Clinical	2017
GCA 016728955.1	China	Clinical	2017
GCA 900407325.1	Tanzania	Clinical	2015
GCA 016729435.1	China	Clinical	2017
GCA 019971815.1	Japan	Unknown	2019
GCA_019972495.1	Japan	Unknown	2018
GCA_019972455.1	Japan	Unknown	2018
GCA 014169235.1	Japan	Environment	2018
GCA 000721855.1	Malaysia	Environment	2014
GCA 017280155.1	China	Clinical	2016
GCA 019971735.1	Japan	Unknown	2019
GCA 019971695.1	Japan	Unknown	2019
GCA 019971175.1	Japan	Unknown	2018
GCA 019971155.1	Japan	Unknown	2018
GCA 019971615.1	Japan	Unknown	2019
GCA 019971195.1	Japan	Unknown	2018
FAHZZU2447	China	Clinical	2019
GCA 019395105.1	Thailand	Unknown	2019

GCA 016729825.1	China	Clinical	2017
GCA 016729935.1	China	Clinical	2017
GCA 016729465.1	China	Clinical	2017
GCA 019971775.1	Japan	Unknown	2019
GCA 019971875.1	Japan	Unknown	2019
GCA 019971835.1	Japan	Unknown	2019
GCA 019971995.1	Japan	Unknown	2019
GCA 019972635.1	Japan	Unknown	2019
GCA 019972035.1	Japan	Unknown	2019
GCA 019971635.1	Japan	Unknown	2019
GCA 019972015.1	Japan	Unknown	2019
GCA 019971915.1	Japan	Unknown	2019
GCA 019972715.1	Japan	Unknown	2019
GCA 019972755.1	Japan	Unknown	2019
GCA 016861425.1	Japan	Unknown	2019
GCA 019971935.1	Japan	Unknown	2019
GCA 019971715.1	Japan	Unknown	2019
GCA 019971955.1	Japan	Unknown	2019
GCA 019972615.1	Japan	Unknown	2019
GCA 019972575.1	Japan	Unknown	2019

GCA 019971975.1	Japan	Unknown	2019

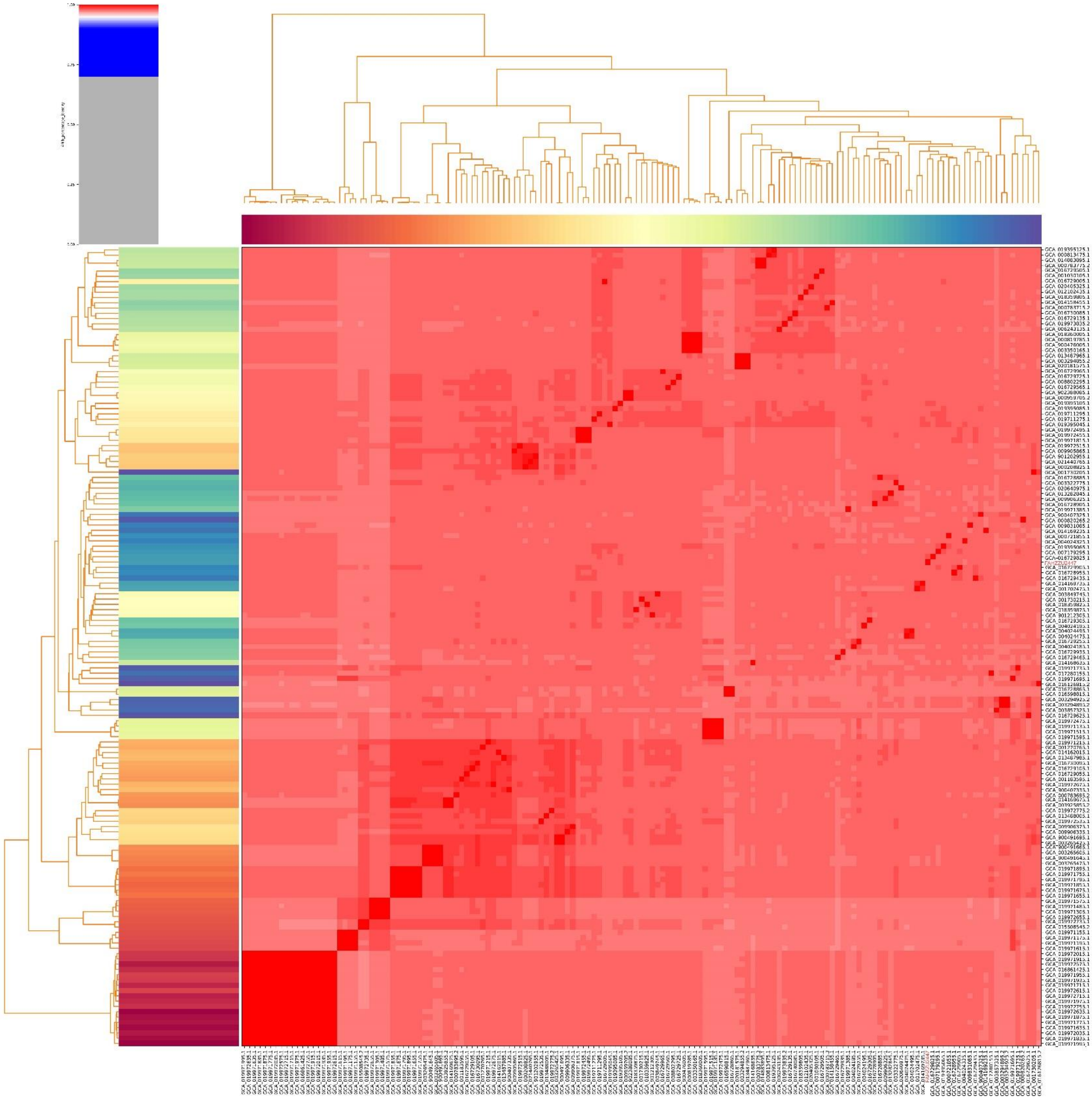

Figure S1. The ANI analysis of *A. caviae* FAHZZU2447. The reference genomes were downloaded from the NCBI database.

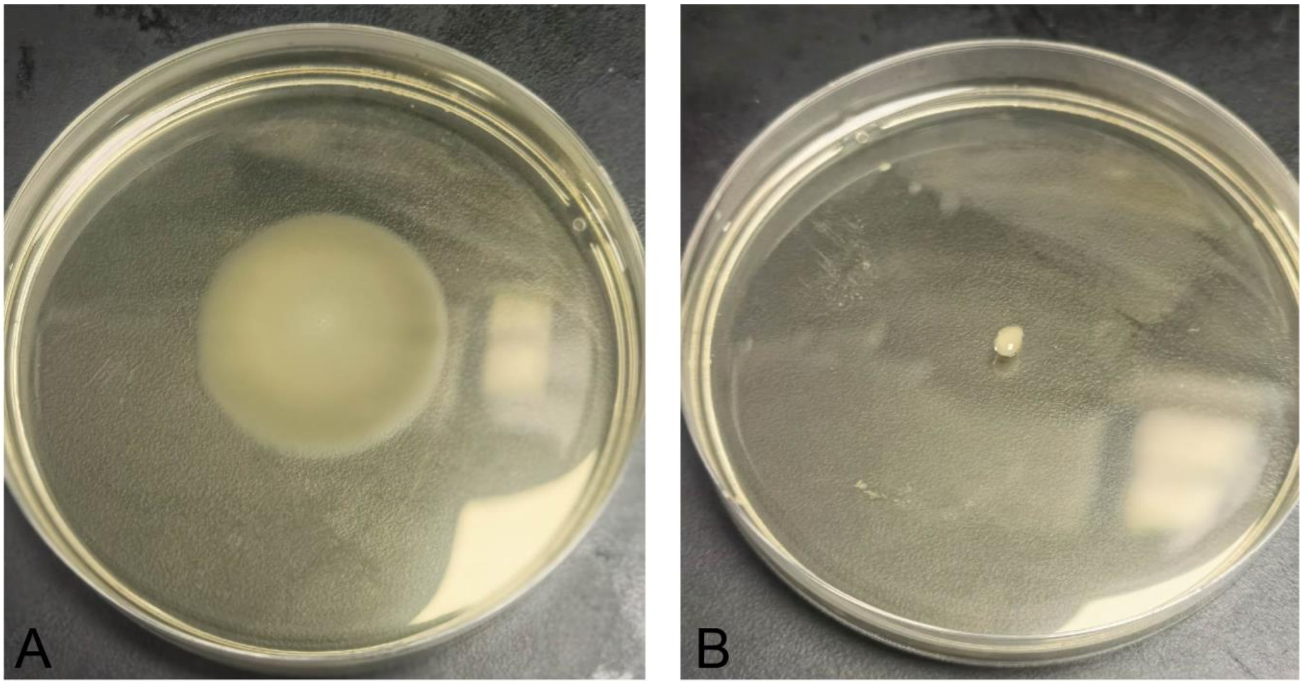

Figure S2. Motility phenotype of *A. caviae* FAHZZU2447 (A). The non-motile *Klebsiella pneumoniae* was used as negative control (B).

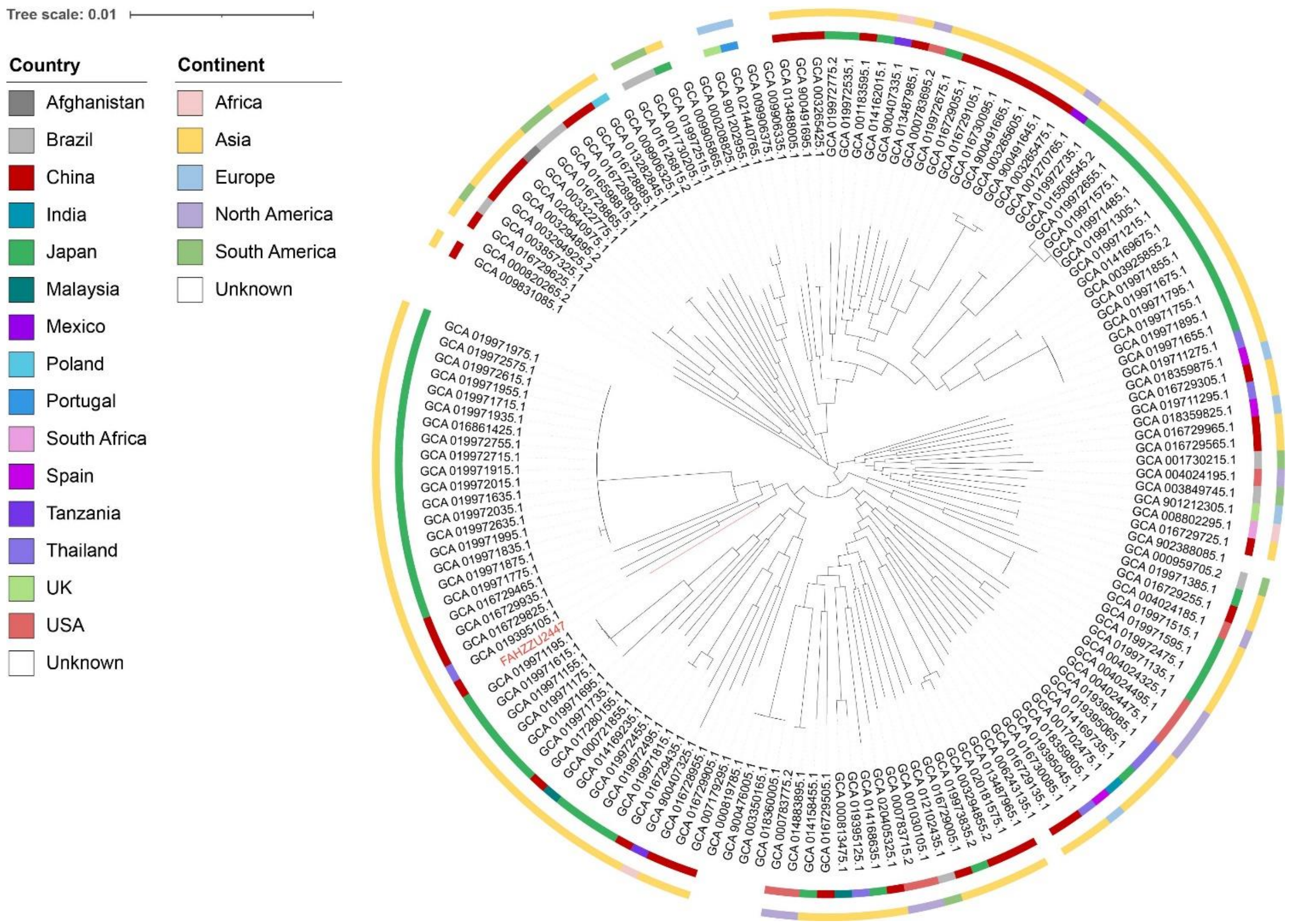

Figure S3. A phylogenetic tree showing *A. caviae* strain FAHZZU2447 along with all additional *A. caviae* genomes are publicly available in the NCBI Genome database. The countries and continents of isolates are presented by different colors.
